# Yue-bi-tang attenuates adriamycin-induced nephropathy edema through decreasing renal microvascular permeability via inhibition of the Cav-1/ eNOS pathway

**DOI:** 10.3389/fphar.2023.1138900

**Published:** 2023-05-09

**Authors:** Tingting Li, Su Cheng, Lin Xu, Pinglan Lin, Minghai Shao

**Affiliations:** ^1^ Department of Nephrology, Shuguang Hospital Affiliated to Shanghai University of Traditional Chinese Medicine, Shanghai, China; ^2^ Key Laboratory of Liver and Kidney Diseases, Ministry of Education, Shanghai University of Traditional Chinese Medicine, Shanghai, China; ^3^ TCM Institute of Kidney Disease, Shanghai University of Traditional Chinese Medicine, Shanghai, China; ^4^ Shanghai Key Laboratory of Traditional Chinese Clinical Medicine, Shanghai University of Traditional Chinese Medicine, Shanghai, China

**Keywords:** Yue-Bi-tang, microvascular permeability, nephrotic syndrome, Cav-1/eNOS, edema

## Abstract

Edema is one of the most typical symptoms of nephrotic syndrome. Increased vascular permeability makes a significant contribution to the progression of edema. Yue-bi-tang (YBT) is a traditional formula with excellent clinical efficacy in the treatment of edema. This study investigated the effect of YBT on renal microvascular hyperpermeability-induced edema in nephrotic syndrome and its mechanism. In our study, the content of target chemical components of YBT was identified using UHPLC-Q-Orbitrap HRMS analysis. A nephrotic syndrome model was replicated based on male Sprague-Dawley rats with Adriamycin (6.5 mg/kg) by tail vein injection. The rats were randomly divided into control, model, prednisone, and YBT (22.2 g/kg, 11.1 g/kg, and 6.6 g/kg) groups. After 14 d of treatment, the severity of renal microvascular permeability, edema, the degree of renal injury, and changes in the Cav-1/eNOS pathway were assessed. We found that YBT could regulate renal microvascular permeability, alleviate edema, and reduce renal function impairment. In the model group, the protein expression of Cav-1 was upregulated, whereas VE-cadherin was downregulated, accompanied by the suppression of p-eNOS expression and activation of the PI3K pathway. Meanwhile, an increased NO level in both serum and kidney tissues was observed, and the above situations were improved with YBT intervention. It thus indicates YBT exerts therapeutic effects on the edema of nephrotic syndrome, as it improves the hyperpermeability of renal microvasculature, and that YBT is engaged in the regulation of Cav-1/eNOS pathway-mediated endothelial function.

## Introduction

Nephrotic syndrome (NS), one cause of end-stage kidney disease, is characterized by massive proteinuria with peripheral edema, hypoalbuminemia, and hypercholesterolemia as its main clinical features (Hull and Goldsmith, 2008). Although edema is the cardinal manifestation of NS at the onset of illness, its mechanisms and therapeutic strategies of edema have long been studied and have sparked heated debates (Siddall and Radhakrishnan, 2012; Meena and Bagga, 2020). Microvascular hyperpermeability is critical in the pathophysiology of nephrotic edema associated with NS (Rostoker et al., 2000; Siddall et al., 2017). As a consequence, attenuating renal microvascular hyperpermeability might be a key element in preventing edema in the progression of NS.

In addition to the transcellular and paracellular routes, vascular permeability could also occur via caveolae-mediated transcellular pathways (Bauer et al., 2005). Caveolae, 50–100 nm flask-shaped invaginations of the plasma membrane, are abundant in endothelial cells with approximately 73 caveolae per μm^2^ of the endothelium (Xu et al., 2018). Caveolin-1 (Cav-1), the main component of caveolae on the plasma membrane, is a 21–22 KD protein with multiple functions, including plasma protein transport and sorting of signaling molecules such as endothelial nitric oxide synthase (eNOS) and nonreceptor tyrosine kinases (Zhang et al., 2020; Guerit et al., 2021; Luo et al., 2021). Cav-1 inactivates nitric oxide (NO) signaling by binding and inhibiting eNOS, thus regulating vascular permeability and angiogenesis (Frank et al., 2003). Garrean et al. confirmed that the knockout of Cav-1 and the activation of eNOS could attenuate lung microvascular hyperpermeability and edema formation in mice (Garrean et al., 2006). However, the change of the expression in Cav-1 and the activation of eNOS in NS could be attractive targets to ameliorate edema.

Yue-bi-tang (YBT), a popular traditional Chinese herbal medicine, has been widely used clinically to treat edema in NS. It consists of five Chinese herbs: *Ephedra sinica Stapf* (Ma Huang), *Zingiber officinale Roscoe* (Sheng Jiang), *Gypsum Fibrosum* (Shi Gao), *Ziziphus zizyphus* (Da Zao), and *Glycyrrhizae Radix et Rhizoma* (Gan Cao), aiming to “dispel the wind and disperse lung-qi for diuresis.” YBT can effectively relieve edema from kidney injuries such as acute glomerulonephritis (Hu et al., 2020). However, the specific molecular mechanisms by which YBT alleviates Adriamycin-induced nephropathy edema remain unknown, which has been limiting its wider use.

The goal of this study is to investigate whether YBT could alleviate Adriamycin-induced nephropathy edema and reduce renal microvascular permeability by inactivating the Cav-1/eNOS pathway.

## Materials and methods

### Animals and drugs

Male Sprague–Dawley (SD) rats (weighing 220 ± 20g) were purchased from Shanghai Bikai Laboratory Animal Technology Co., Ltd. [number: SCXK (Hu) 2018-0006]. All the rats used in the experiments were housed under standard temperature (23°C ± 3°C) and humidity (55% ± 15%) with a 12 h light/12 h dark cycle in SPF condition while being fed with water and food as standard in the Experimental Animal Center of Shanghai University of TCM. The animal study was reviewed and approved by the Animal Ethics Committee of Shanghai University of Traditional Chinese Medicine (PZSHUTCM220725025).

YBT consists of *Ephedra* 18 g, *Ginger* 9 g, *Gypsum* 24 g, *Fructus Ziziphi Jujubae* 9 g, and *Liquorice* 6 g. The raw herbs for the preparation of YBT were obtained from Shanghai Kangqiao Chinese Medicine Tablet Co., Ltd. (Shanghai, China). All materials were soaked with distilled water 1 time for 30 min and then boiled for 2 h. The medicinal residue from the first extraction was filtered for the second extraction with the same extracting condition. After repeating three times, the mixture of the filtrates was enriched to a concentration of 2.1 g of raw materials per milliliter (w/v). Prednisone was purchased from Guangdong Huanan Pharmaceutical (Guangdong, China). Prednisone was dissolved and diluted in a saline solution with a concentration of 2.5 mg/mL.

### UHPLC-Q-orbitrap HRMS analysis of YBT

The aqueous extract of YBT was treated with a 0.22 μm filter membrane for UHPLC-Q-Orbitrap HRMS analysis. The fingerprints of YBT extracts were obtained by ultra-high-performance liquid chromatography (UHPLC-Q-Orbitrap HRMS, Thermo Fisher Scientific Inc., Grand Island, NY, United States) which was Thermo Fisher Dionex Ultimate 3000 using Chromeleon 7.2 software for operation. UHPLC conditions: The sample chamber was protected from light, the temperature was set at 10°C, and the column temperature was set at 40°C. The separation was performed through an ACQUITY UPLC BEH C18 column (2.1 × 100 mm, 1.7 μm) using a mobile phase of methanol and 0.1% formic acid delivered in gradient elution at a flow rate of 0.3 mL/min: 0-2 min, 4% methanol; 2-6 min, 4%-12% methanol; 6-38 min, 12%-70% methanol; 38- 38.5 min, 70% methanol; 38.5-39 min, 70%-95% methanol; 39-43 min, 95% methanol; 43-45 min, 4% methanol. The injection volume was 2 μL. Q-Orbitrap HRMS conditions: The UHPLC tandem quadrupole/electrostatic field orbital trap mass spectrometry was equipped with an electrospray ion source, and data were analyzed and acquired through Xcalibur 4.1. The ion source was used in positive and negative ion modes, and the optimized mass spectrometry parameters included: capillary temperature of 325°C; sheath gas (N_2_) flow rate of 45 arb; auxiliary gas (N_2_) flow rate of 8 arb; sweep gas flow rate of 0 arb; spray voltage of 2.5 kV (negative ions) and 3.2 kV (positive ions); transmission voltage of 50 V; and auxiliary gas heater temperature of 300°C. Full scan mode was used: scan range 80-1200 m*/z*. Full scan mode was adopted: scan range 80-1200 m/z. Quantitative and qualitative analysis were conducted on ephedrine [(M + H]^+^, *m/z* 166.12262)], pseudoephedrine [(M + H)^+^, *m/z* 166.12262], 6-gingerol [(M + Na)^+^, *m/z* 317.17206], and 8-gingerol [(M + Na)^+^, *m/z* 345.20343], 10-gingerol [(M + Na)^+^, *m/z* 373.23483], glycyrrhizin [(M-H)^-^, *m/z* 417.11877], and glycyrrhetinic acid [(M-H)^-^, *m/z* 821.396,427]. Maximum injection time (IT): 200 m; scan resolution 70,000 FWHM (m/z/s); automatic gain control (AGC) target: 1.0e^6^.

### Groups and drug administration

After 1 week of acclimatization, apart from the 10 rats randomized into the control group with saline (1.0 mL/100 g), we intravenously administered the rest with Adriamycin (ADR, 6.5 mg/kg dissolved in saline, Shenzhen Main Luck Pharmaceuticals Inc.) to establish a NS rat model (Teng et al., 2017). In other words, a nephritis model was established by injecting Adriamycin in the caudal vein only once. Two weeks after injection, the successful modeling rats with higher 24 h proteinurias were randomly divided into five groups (10 animals in each group): model group, prednisone (5.0 mg/kg, Guangdong Huanan Pharmaceutical, China) group, and YBT group (YBT 22.2 g/kg, 11.1 g/kg and 6.6 g/kg). The dosage of YBT was calculated according to the equivalent dosage formula of rats and the adult weight of 65 kg. The gavage dosages of YBT and prednisone were also chosen based on the clinical dosage and the result of our preliminary experiment. All the rats were administered distilled water (control group and model group), prednisone, or YBT by means of oral gavage oral gavage once a day for 2 weeks.

### Blood and uric indexes analysis

To assess urinary protein levels, the 24 h urine was acquired on days 0, 14, 21, and 28 with a metabolic cage, respectively. At the end of interventions, blood, and renal tissues were collected. The levels of 24 h urine protein (24 h UTP), albumin (ALB), total cholesterol (TC), serum creatinine (Scr), urea nitrogen (BUN), and hemoglobin (HB) were detected by the automatic biochemical analyzer (AU680, Beckman Coulter, United States) in Clinical Laboratory of Shuguang hospital.

### Kidney and skin wet-to-dry (W/D) weight ratio

The skin and kidneys were removed, washed with physiological saline, and the surface liquid was blotted out with filter paper, weighed wet, and recorded. After putting them into a constant temperature incubator at 50°C for drying, the dry weights were weighed after 72 h. Based on the wet-to-dry weight (W/D) ratio, the degree of kidney and skin edema was evaluated.

### Evans blue staining

Referring to related studies (Aman et al., 2012; Koning et al., 2018), renal microvascular permeability can be evaluated using the Evans Blue dye extravasation method. The 2% Evans Blue (EB) (Sigma-Aldrich, F9037, United States) was injected into the tail vein at 1 mL/kg, circulated *in vivo* for 1 h and perfused with PBS to remove the intravascular Evans Blue dye. Kidney tissues with 100 mg of Evans blue dye were extracted by putting the tissues in formamide (Sigma-Aldrich, F9037, United States) at 1 g/mL. After incubating at 60°C for 24 h, we detected the OD value at the wavelength of 620 nm using the microplate reader (Cytation 3, Biotek, United States)

### Western blot analysis

The total proteins of kidney tissues were extracted with a RIPA lysis buffer including protein hydrolase and phosphatase inhibitors. Protein concentration was calculated using a BCA protein analysis kit (Beyotime Biotech, P0010, China) following the instructions of the manufacturer. Protein samples were separated by 8% or 1% SDS-PAGE electrophoresis for 90 min, transferred to polyvinylidene difluoride membranes (Millipore, United States) (100 V, 1-2 h), and blocked in 5% non-fat milk in a shaker for 2 h at the room temperature. Afterwards, the membranes were incubated overnight at 4°C with corresponding primary antibodies as follows: anti-Cav-1 (CST, 3267, United States), anti-VE-cadherin (Santa, sc-9989, United States), anti-p-eNOS (Affinity, AF3247, China), anti-p-AKT (CST, 4060, United States), anti-AKT (CST, 4691, United States), anti-PI3K (CST, 4292, United States) and anti-Gapdh (Proteintech, 60004-1-Ig, United States). The binding of the primary antibody was detected by the ECL method (180-501 ECL, Tanon, China) using horseradish peroxidase-conjugated secondary antibodies (goat anti-rabbit IgG, A0208 or goat anti-mouse IgG, A0216, Beyotime Biotech, China). Quantitative analysis was performed using ImageJ software (NIH, Bethesda, MD, United States).

### Histological staining of renal tissue

Kidney tissues were fixed in a 4% paraformaldehyde solution, dehydrated in graded alcohol, embedded in paraffin, and cut into 3 μm slices. After that, hematoxylin-eosin (H&E) staining, Masson’s trichrome, and periodic acid-Schiff (PAS) staining were performed according to the standard method. The pathological changes in the glomerulus were observed under light microscopy (Nikon Eclipse 80i, Japan).

### Immunofluorescence (IF) and immunohistochemistry (IHC) analysis

After dewaxing the kidney samples with xylene, the antigen was repaired and closed with 3% BSA at room temperature for 30 min, and the primary antibody Cav-1 (1:100, CST), VE-cadherin (1:50, Santa) were incubated overnight at 4°C, protected from light. Then the sections were washed twice with PBS, incubated in fluorescent secondary antibody for 1 h at 37°C, protected from light, washed with PBS, and stained with DAPI for 5 min. The positive expressions were observed with a fluorescence microscope (Nikon Eclipse80i, Japan) at ×400 magnification. For immunohistochemistry staining, kidney tissues were paraffin sections dewaxed to water, and antigens were repaired. After blocking endogenous peroxidase, anti-p-eNOS (1:50, Affinity, AF3247, China) was added and then incubated overnight at 4°C. Sections were rinsed 3 times in PBS. Then secondary antibodies were incubated at room temperature for 50 min. DAB staining kit (Suokeer, biotech, Nanjing, China) was used for color enhancement and observed under bright field microscopy (Nikon Eclipse 80i, Japan) at ×400 magnification, with positive expression as brownish-yellow particles. Four areas randomly selected in each section were photographed and analyzed by Image-Pro plus version 6.0.

### Evaluation of NO levels

We quantified the NO level in rats’ serum and kidney tissues according to the kit specifications (Nanjing Jiancheng Bioengineering Institute, A013-2-1, China), and measured the absorbance of each sample at 550 nm on an enzyme calibrator.

### Statistical analysis

All data were expressed as mean ± standard deviation (SD) and analyzed by one-way analysis of variance with LSD-t multiple comparisons using SPSS software (version 26.0, SPSS Inc., Chicago, United States). *p <* 0.05 was considered statistically significant.

## Results

### UHPLC-Q-orbitrap HRMS analysis of YBT

**FIGURE 1:**
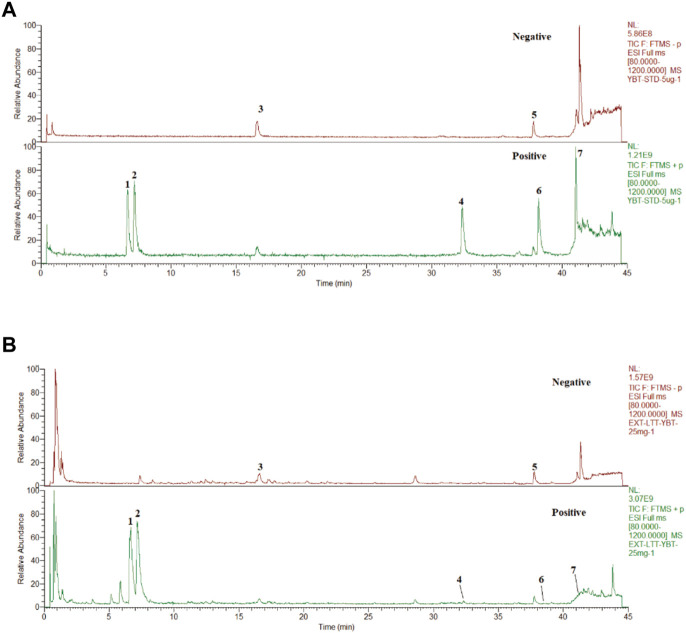
YBT quality control by UHPLC-Q-Orbitrap HRMS analysis. **(A)** The fingerprint chromatograms of the extract of mixture reference standards in positive and negative ion mode by UHPLC-Q-Orbitrap HRMS.1 = Ephedrine, 2 = pseudoephedrine, 3 = liquidity, 4 = 6-gingerol,5 = glycyrrhizic acid,6 = 8-gingerol,7 = 10-gingerol. **(B)** The fingerprint chromatograms of the extract of YBT in positive and negative ion mode by UHPLC-Q-Orbitrap HRMS. 1 = Ephedrine, 2 = pseudoephedrine, 3 = liquiritin, 4 = 6-gingerol, 5 = glycyrrhizic acid, 6 = 8-gingerol, 7 = 10-gingerol.

After assessing the quality of YBT by UHPLC-Q-Orbitrap HRMS, the content of target chemical components of YBT was identified. The total ion flow diagrams of YBT extract and mixed control in positive and negative ion modes were shown in Figure 1. And the contents of ephedrine, pseudoephedrine, glycyrrhizin, 6-gingerol, glycyrrhetinic acid, 8-gingerol, and 10-gingerol as the target compounds in YBT extract were measured as 22.66 μg/mL, 18.14 μg/mL, 7.04 μg/mL, 0.50 μg/mL, 11.90 μg/mL, 0.0029 μg/mL and 0.0015 μg/mL, respectively (Table 1).

**TABLE 1:** The content of target chemical components of YBT.

Peak serial number	Name	Molecular formula	Content (μg/mL)
1	Ephedrine	C_10_H_15_NO	22.66
2	Pseudoephedrine	C_10_H_15_NO	18.14
3	Liquiritin	C_21_H_22_O_9_	7.04
4	6-gingerol	C_17_H_26_O_4_	0.50
5	Glycyrrhizic acid	C_42_H_62_O_16_	11.90
6	8-gingerol	C_43_H_32_O_20_	0.0029
7	10-gingerol	C_21_H_34_O_4_	0.0015

### YBT treatment improved edema and microvascular permeability in rats with the NS model

**FIGURE 2:**
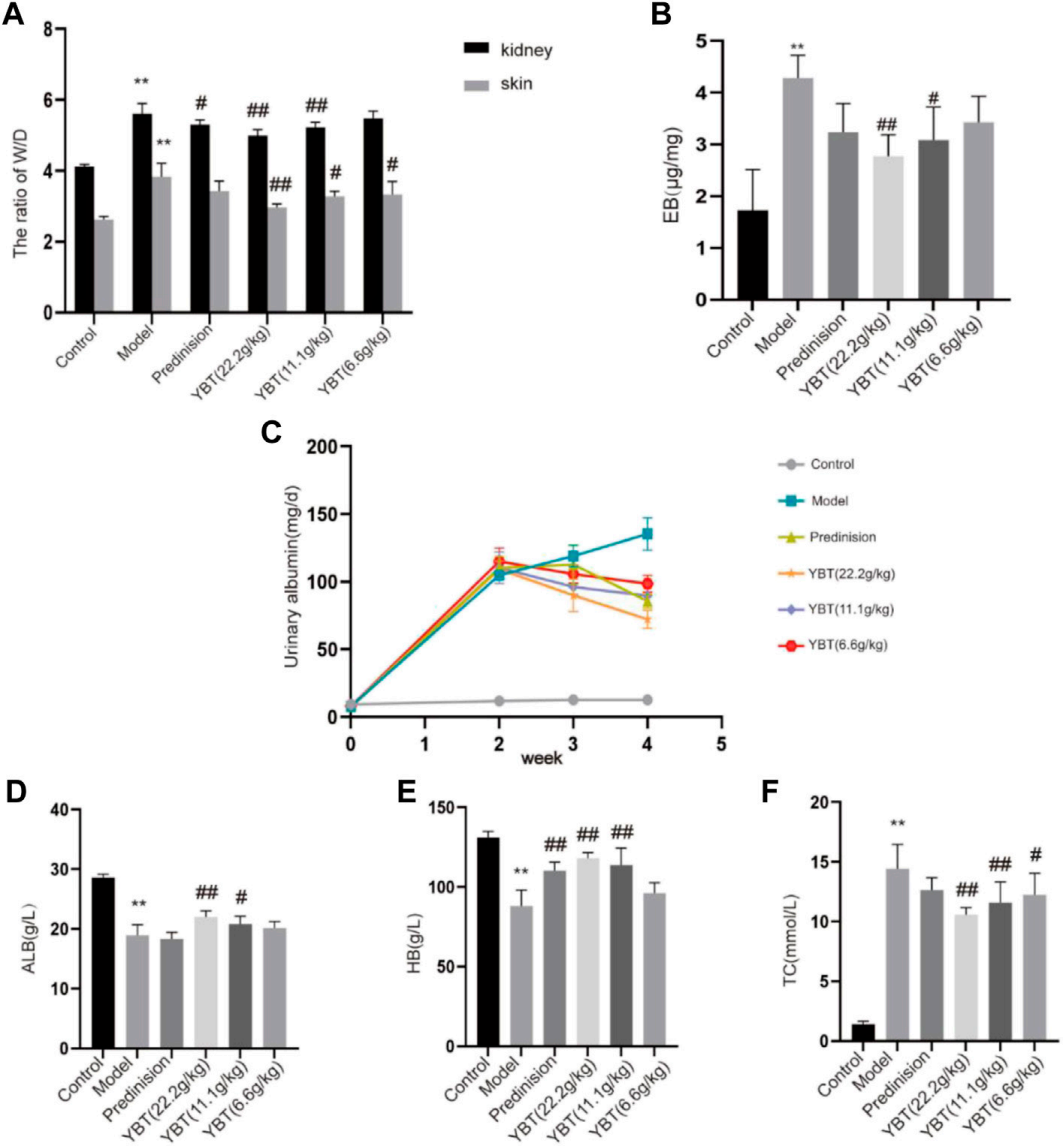
Effect of YBT on symptoms and renal vascular function in nephrotic syndrome. **(A)** The skin and kidney W/D weight ratio in each group (*n* = 6). **(B)** Evans Blue dye extravasation (*n* = 4). **(C)** 24 h urine protein (24 h UTP): Pre-modeling, 14 days post-modeling, 7 days on medication, 14 days on medication (*n* = 6). **(D–F)**: The level of albumin (ALB), hemoglobin (HB), and total cholesterol (TC) in each group (*n* = 6). Data present means ± SD, compared with control, *p < 0.05, **p < 0.01, compared with model, #*p* < 0.05, ##*p* < 0.01.

The occurrence of edema was associated closely with vascular leakage (Fantin et al., 2017). In this study, we used the ADR-induced NS (ADR-NS) model to investigate the effect of YBT on the alleviation of edema and microvascular hyperpermeability. As shown in Figure 2, compared with the control group, the W/D weight ratio of skin and kidney and the content of Evans Blue dye of tissues in the model group was significantly increased (Figures 2A,B); compared with the model group, YBT, and prednisone decreased the W/D weight ratio of skin and kidney and the content of Evans Blue dye of tissues. Meanwhile, compared with the control group, 24 h UTP was raised in the model group. However, YBT and prednisone gradually reduced 24 h UTP after 7 d and 14 d of treatment (Figure 2C). In addition, HB and ALB decreased accompanied by TC increased in rats with an ADR-induced NS model compared with the control group, which were reversed by the YBT therapy and were improved with the high dose of YBT (Figures 2D–F). In conclusion, these findings provided strong evidence that YBT could treat not only edema in nephrotic syndrome by altering microvascular permeability but also other symptoms of nephrotic syndrome.

### YBT reduced renal injury in the NS rat model

**FIGURE 3:**
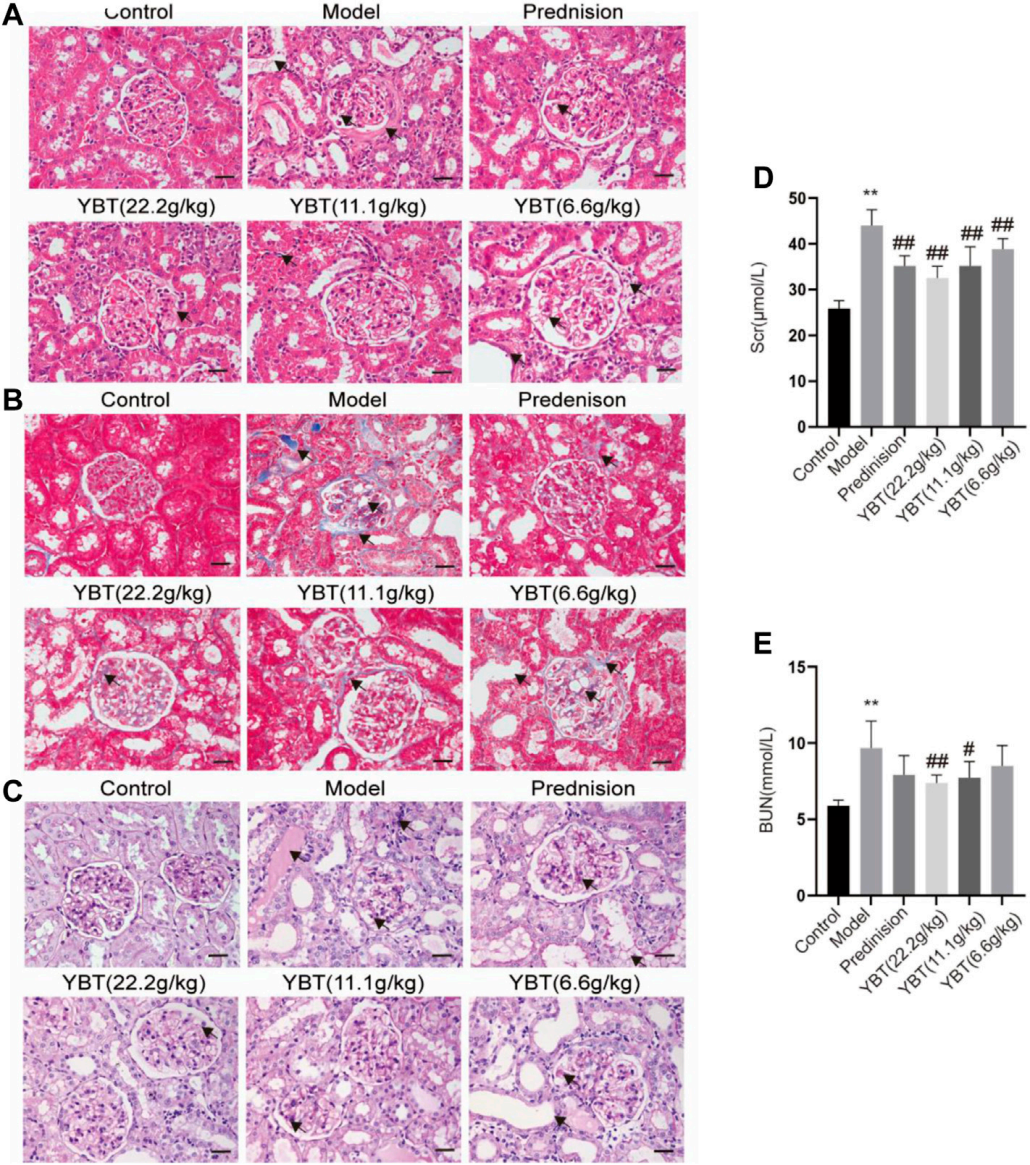
Effects of YBT on renal function and renal pathology in ADR-NS rats model. **(A)** HE staining of rat kidney tissues. Magnification, ×400. Scale bar, 25 µm. **(B)** Masson’s trichrome staining of rat kidney tissues. Magnification, ×400. Scale bar, 25 µm. **(C)** PAS staining of rat kidney tissues. Magnification, ×400. Scale bar, 25 µm. **(D)** and **(E)** The level of serum creatinine (Scr) and urea nitrogen (BUN) (*n* = 6). Values represent mean *±* SD, compared with control, *p < 0.05, **p < 0.01, compared with model, #*p* < 0.05, ##*p* < 0.01.

To explore the effect of YBT on kidney injury, serum biochemical analysis, HE staining, Masson staining, and PAS staining of kidney tissues were performed on rats. We first tested the levels of serum creatinine and blood urea nitrogen. It was found that the levels of both serum creatinine and blood urea nitrogen in the model group rose compared with the control group. Meanwhile, they could be downregulated via YBT treatment, and the higher the dose the better (Figures 3D,E). Histological analysis of the kidneys showed that the glomeruli of the control group were intact. In contrast, the glomeruli of the model group demonstrated glomeruli adhesion, thickened basement membrane, vacuolation of endothelial cells, collagen deposition by Masson staining, and glycogen deposition by PAS staining. It was observed that the epithelial cells of the renal tubules were vacuolated and deformed, the lumen was enlarged, and the renal interstitium was edematous and infiltrated with inflammatory cells. Significant improvements were observed in renal pathology after YBT treatment (Figures 3A–C).

YBT improved the vascular filtration barrier possibly by inhibiting Cav-1 expression in the ADR-NS model.

**FIGURE 4:**
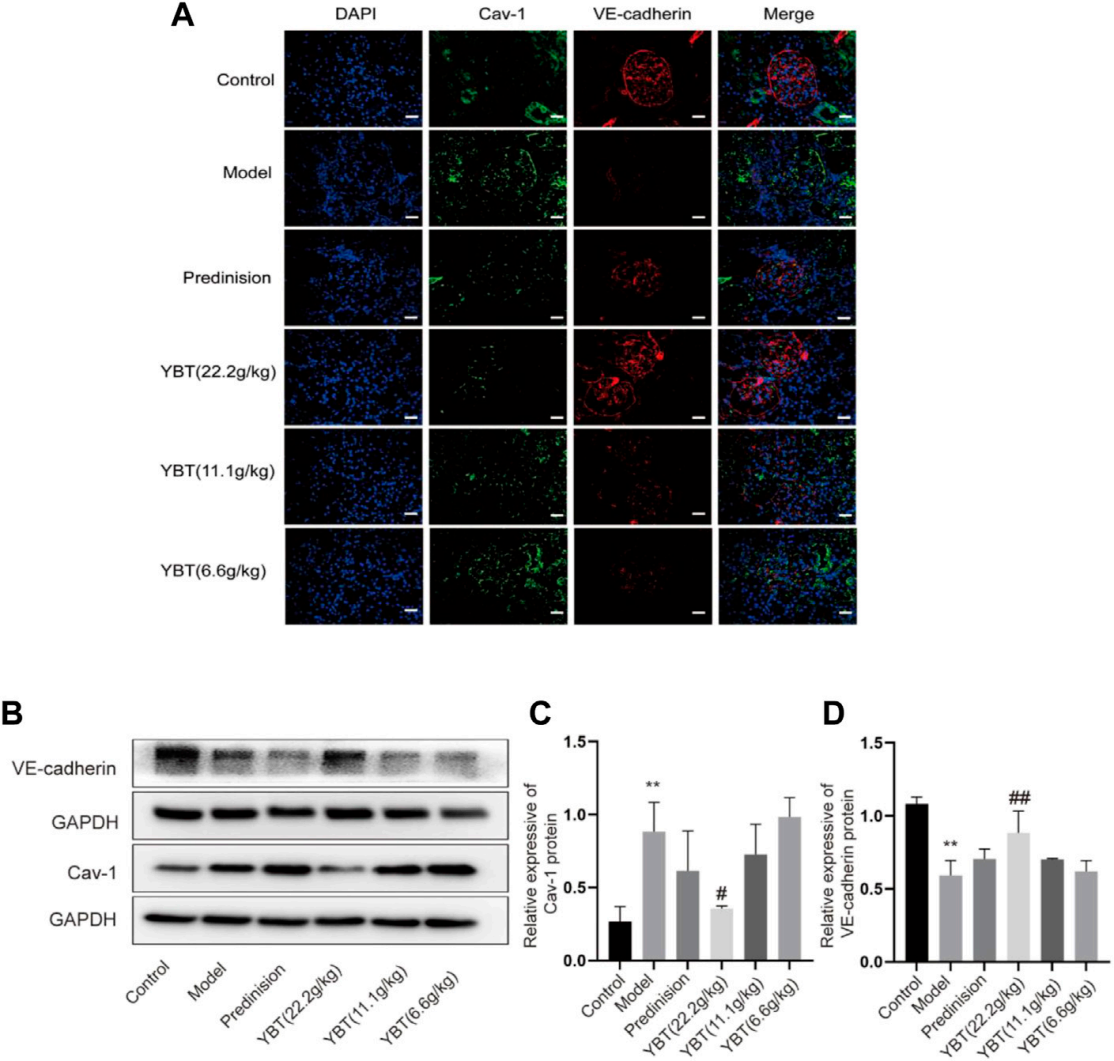
Effects of YBT on Cav-1-mediated endothelial barrier function in ADR-NS rat model. **(A)** Representative images of immunofluorescence staining of Caveolin-1(Cav-1, green), VE-cadherin (red), and DAPI (blue). Magnification, ×400. Scale bar, 25 µm. **(B)** Western blot analysis of Cav-1 and VE-cadherin expression (*n* = 3). **(C)** and **(D)** Quantitation of Cav-1 and VE-cadherin expression in each group (*n* = 3). Date represent mean *±* SD, compared with control, *p < 0.05, **p < 0.01, compared with model, #*p* < 0.05, ##*p* < 0.01.

Inhibition of Cav-1 expression could be an effective means of limiting vascular injury by preventing increased transcellular albumin permeability and stabilizing the endothelial junction barrier (Sun et al., 2009; Smit et al., 2018). As shown in the immunohistochemical fluorescence analysis of the kidney tissues, there was an increased Cav-1 expression and a declined VE-cadherin expression in glomerular micro-vessels compared with the control group, indicating that the Cav-1 expression was associated with the vascular barrier. YBT treatment could suppress Cav-1 expression and activate VE-cadherin expression in a dose-dependent manner (Figure 4A). Moreover, the immunoblotting examination demonstrated that the expression of Cav-1 protein elevated, and the VE-cadherin protein expression dropped in the ADR-NS model group compared with those of the control group (Figures 4B–D). Meanwhile, compared with the model group, YBT suppressed the protein expression of Cav-1 and stimulated the VE-cadherin. Taken together, these experimental results suggested that the YBT treatment for edema in nephrotic syndrome achieved the potential effect in part by modulating the Cav-1 expression to regulate albumin transport across membranes and intercellular adhesion junctions, thereby maintaining the normal barrier function of vascular endothelial cells.

### YBT treatment regulated the expression of Cav-1 associated with altered PI3K/AKT and eNOS signaling

**FIGURE 5:**
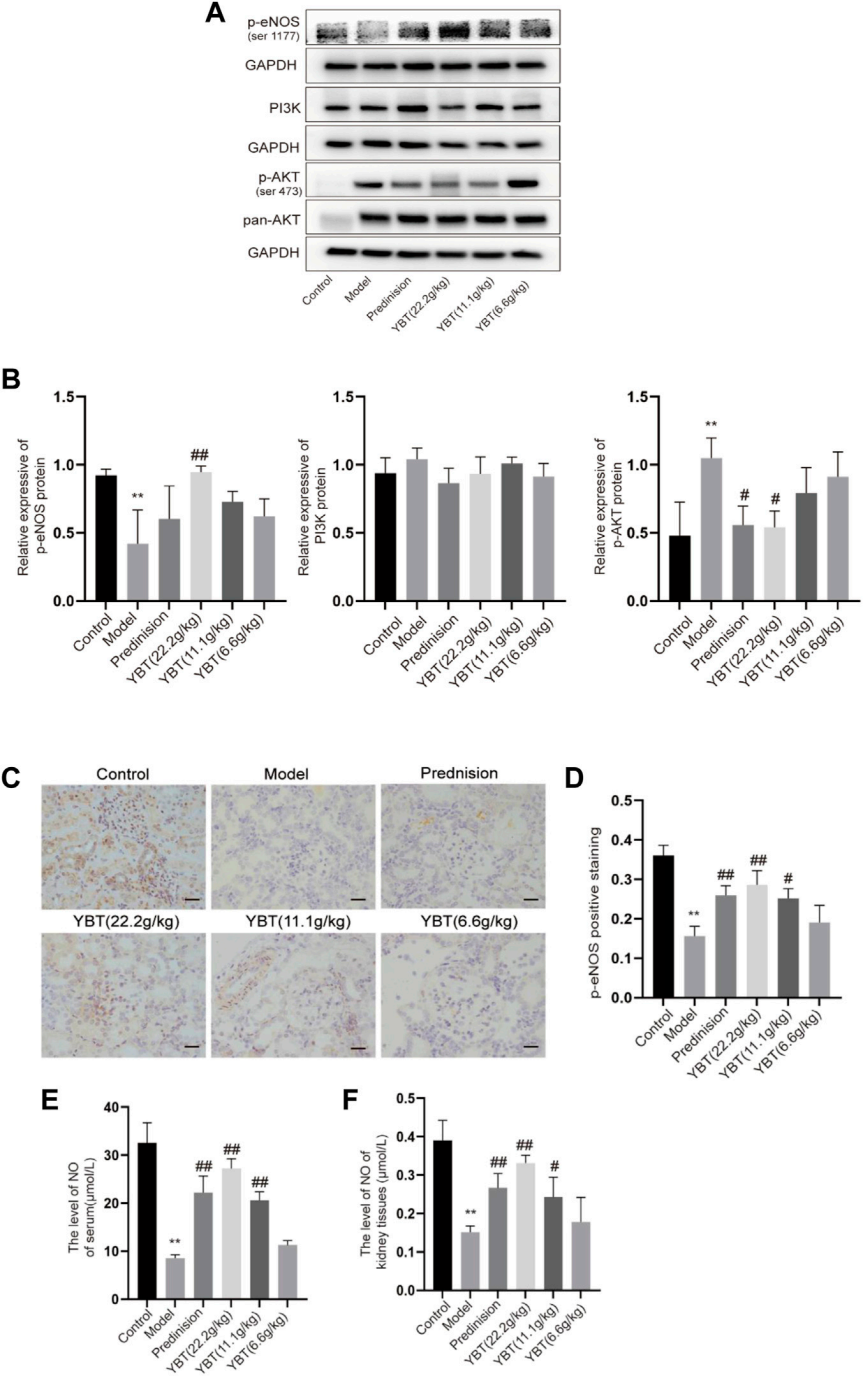
Effects of YBT on other mechanisms associated with Cav-1 in ADR-NS rat model. **(A)** Western blot analysis of p-eNOS, PI3K, and p-AKT expression (*n* = 3). **(B)** Quantitation of p-eNOS, PI3K, and p-AKT expression in each group (*n* = 3). **(C)** Immunohistochemistry of p-eNOS, Magnification, ×400. Scale bar: 25 μm (*n* = 3). **(D)** Semiquantitative analysis of p-eNOS positive staining (*n* = 3). **(E)** The level of NO of serum per group (*n* = 4). **(F)** The level of NO of kidney tissues per group (*n* = 4). Results represent mean *±* SD, compared with control, *p < 0.05, **p < 0.01, compared with model, #*p* < 0.05, ##*p* < 0.01.

Cav-1 is critical for NO production via inactivating eNOS. Both Cav-1 and eNOS are modulated by PI3K/AKT signaling (Chen et al., 2021; Chen J. et al., 2022). To further understand the effector molecules of YBT-induced Cav-1 regulation, we performed immunoblotting and immunohistochemical experiments on PI3K/AKT and eNOS signaling, respectively. As shown in Figures 5A,B, immunoblotting experiments revealed that AKT phosphorylation levels were elevated, and eNOS phosphorylation was inhibited in the model group. After YBT treatment, AKT phosphorylation levels fell, and eNOS phosphorylation levels grew, preferably at the high dose of YBT. Besides, there was no significant difference in the change of PI3K expression among the groups. As for immunohistochemistry, the results agreed with Western blot that YBT treatment could upregulate the protein expression of p-eNOS with a high dose of YBT being preferred (Figures 5C,D).

To further determine whether YBT treatment affects microvascular permeability by influencing the level of NO which is a product of eNOS, we measured the NO levels in serum as well as kidney tissues. As shown in Figures 5E,F, compared with the control group, NO levels in both serum and renal tissues were reduced in the model group, which were notably elevated by prednisone and YBT treatment, particularly in the YBT high-dose group. In summary, YBT could regulate the Cav-1/eNOS signaling in the NS rat model.

## Discussion

**FIGURE 6:**
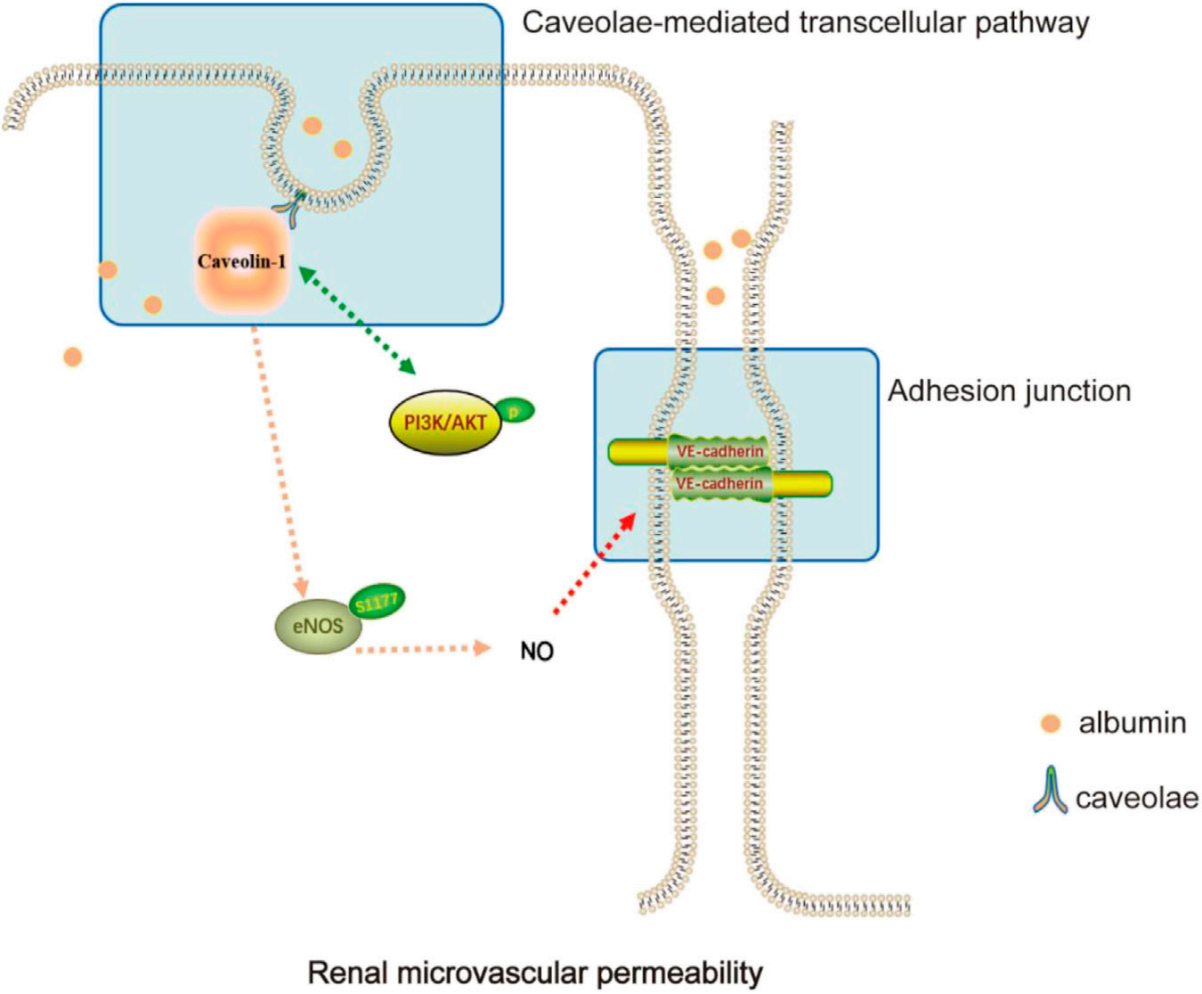
Diagram of the mechanisms involved in the study.

YBT was first described in “Jin-Gui-Yao-Lue,” a classical Chinese medicine work. According to the theory of traditional Chinese medicine, YBT is widely used in modern medicine for edema-like diseases due to its excellent effectiveness in improving proteinuria (Hu et al., 2020). Studies have shown that YBT can reduce proteinuria, modulate renal tissue aquaporins, regulate transient receptor potential ion channels, relieve renal damage and protect renal function in rats with adriamycin nephropathy (Song et al., 2020; Liu et al., 2022). YBT consists of *Ephedra sinica Stapf* (Ma Huang), *Zingiber officinale Roscoe* (Sheng Jiang), *Gypsum Fibrosum* (Shi Gao), *Ziziphus zizyphus* (Da Zao), and *Glycyrrhizae Radix et Rhizoma* (Gan Cao). Studies have shown that *E. sinica Stapf* can exert anti-inflammatory, antioxidant, antiviral, and diuretic effects (Zhang et al., 2018; Chen Y. Q. et al., 2022). Quercetin, an extract of *E. sinica Stapf*, can improve vascular leakage and has a protective effect on vascular endothelial cell damage (Tripathi et al., 2019; Kondo-Kawai et al., 2021). *Zingiber officinale Roscoe* has the effect of regulating apoptosis, immunity and inflammation, and cytoskeletal adhesion (Kiyama, 2020). Its main components have potential benefits in the treatment of diabetic nephropathy, hypertension, metabolic syndrome, and other diseases (Alsherbiny et al., 2019; Tan et al., 2022). 6-Gingerol increases the integrity of the endothelial cell (EC) barrier and the tight junctions between the periphery and periphery of the EC to maintain normal microvascular function (Zhong et al., 2019). In addition, its active ingredients can also promote cholesterol efflux from macrophages, reduce oxidative stress, improve inflammation, and induce autophagy, thus exerting vascular protective effects (Li et al., 2021). *Glycyrrhizae Radix et Rhizoma* can inhibit thrombosis, regulate lipid metabolism, antioxidant, and so on, commonly treated for diabetic nephropathy, liver damage, gastrointestinal ulcer, asthma, and other diseases (Sharifi-Rad et al., 2021). Licorice flavonoids, the main active components of *Glycyrrhizae Radix et Rhizoma*, can inhibit NLRP3-mediated vascular endothelial cell scorching by regulating SIRT6 to enable the normal function of blood vessels (He et al., 2023). In this study, we investigated the ameliorative effect of YBT on edema in nephrotic syndrome and its potential regulatory mechanisms. It was demonstrated that YBT significantly reduced the permeability of renal microvasculature in the ADR-induced NS rat model, thereby alleviating edema in the skin and kidneys. Meanwhile, it was also found that YBT significantly mitigated renal dysfunction, hyperlipidemia, and renal histological damage in the NS rats model. It was worth noting that the above mechanism of YBT action was possibly relevant to the regulation of the Cav-1/eNOS signaling pathway (Figure 6).

Regulating microvascular permeability has a crucial role in promoting the treatment of edema in nephrotic syndrome (Gupta et al., 2018). Udwan et al. reported that capillary permeability was altered, and that water filtration coefficients of paracellular and transcellular pathways were increased, while reflex coefficients to proteins were decreased in the NS rat model (Udwan et al., 2016). Some research findings emphasized that inhibiting capillary leakage improved microcirculatory perfusion and consequently reduced organ edema (Dekker et al., 2018; Koning et al., 2018). Based on these discoveries, modulating capillary permeability is a potential approach to ameliorate edema. In addition, YBT has been proven to be clinically effective in treating NS, especially in improving edema. Hu et al. demonstrated that YBT might reduce pulmonary and renal edema in rats with severe acute pancreatitis via the regulation of water metabolism (Hu et al., 2020). Our data showed skin and kidney edema and increased renal microvascular permeability in rats with adriamycin nephropathy. Figures showed that after 14 days of YBT treatment, the rats’ microvascular permeability decreased, and edema was reduced. Meanwhile, the rat NS model was observed to have 24 h massive proteinuria, hypoalbuminemia, hyperlipidemia, and other typical symptoms of nephrotic syndrome, accompanied by elevated serum creatinine and blood urea nitrogen, while YBT treatment significantly improved such symptoms. Our results evidenced that YBT could ameliorate edema by adjusting microvascular permeability, consequently affecting renal function and reducing renal injury. However, the molecular mechanisms of how regulating microvascular permeability is regulated need to be further defined.

Recent studies have shown that Cav-1 is one of the crucial modulators of vascular permeability (Choi et al., 2016; Parton et al., 2020; Ren et al., 2021). Y. Komarova et al. reported that the transport of albumin across the endothelium involved two alternative routes: a transcellular pathway via caveolae-mediated vesicular transport, or a paracellular pathway via junctions between endothelial cells (Komarova and Malik, 2010). Caveolae-mediated transendothelial albumin transport plays are closely connected with the effects of microvascular hyperpermeability (Chen et al., 2012). One study indicated that Cav-1 potentially played a role in cell-cell adhesion, thereby assisting the regulation of paracellular permeability (Drab et al., 2001). VE-cadherin is a significant component of vascular adhesion junctions and is expressed only in endothelial cells (Vestweber, 2008). In a model where rats had adriamycin nephropathy with elevated endothelial permeability, we observed upregulation of Cav-1 expression and downregulation of VE-cadherin through immunofluorescence and immunoblotting experiments. YBT, especially in high doses, was able to inhibit the expression of Cav-1 and stimulate the expression of VE-cadherin. Consistent with these observations, Zhang et al. reported that in experiments with rats, minor intestinal edema was attenuated because Cav-1 expression was inhibited and VE-cadherin expression was activated in LPS-induced leakage of albumin from small mesenteric veins (Zhang et al., 2014). In summary, YBT could regulate the transcellular and paracellular pathways by affecting Cav-1, thus resulting in reduced renal microvascular leakage of albumin.

In addition to the mechanism we identified above, eNOS is also shown to be involved in the Cav-1-mediated regulation of endothelial cell permeability (Ren et al., 2021). Some studies demonstrated that in cardiovascular and pulmonary diseases, Cav-1 could inhibit eNOS activity, thereby affecting normal angiogenesis and barrier function (Schubert et al., 2002; Sonveaux et al., 2004; Oliveira and Minshall, 2018). NO, derived from eNOS, maintains vascular tone and is essential for normal vascular homeostasis (Bernatchez et al., 2011). Deficient NO production and decreased NO sensitivity would lead to endothelial dysfunction and, ultimately, an imbalance in intravascular homeostasis (Chen et al., 2018). However, the connection between Cav-1 and eNOS has rarely been discussed in studies about glomerular diseases. Meanwhile, it is essential in many fundamental cellular processes to activate PI3K/AKT signaling (Ku et al., 2017; Shu et al., 2022), as it is, simultaneously, a signaling molecule that forms a complex with Cav-1 and is involved in regulating vascular function (Nag et al., 2017; Yang et al., 2017). Given these facts, Cav-1/eNOS and PI3K/AKT pathways may play an important role in regulating glomerular hyperpermeability. To further confirm the ties between Cav-1 and eNOS in glomerular hyperpermeability, we examined the expression of eNOS phosphorylation and NO content. As the data demonstrated, in a state of renal microvascular hyperpermeability, p-eNOS expression was suppressed both in serum and in the kidney, where the production of NO was inhibited, whereas PI3K/AKT signaling was activated. Fortunately, YBT could improve microvascular permeability by stimulating the expression of eNOS phosphorylation, restricting the activation of AKT, enhancing the production of NO, maintaining vascular tone, and improving microvascular permeability.

Our study emphasized is the first exploration in the untouched field of how YBT reduces edema in nephrotic syndrome by modulating vascular permeability. This study focuses on the effects that YBT brings to the regulation of caveolae-mediated transcellular pathways and intercellular adhesion links in endothelial cells. Regretfully, however, assays that we conducted revealed that microvascular skin permeability also varied with the situation. For operational reasons, we failed to obtain valid data support. In the next step, we will continue this experiment to demonstrate our hypothesis that YBT can improve edema in nephrotic syndrome by regulating systemic microvascular permeability.

In conclusion, we believe that YBT can lessen the leakage of albumin through vesicular transport via the Cav-1/eNOS signaling pathway, increase intercellular adhesion junctions, and maintain the permeability of renal microvessels, thus proposing a promising option for treating nephrotic edema and relieving renal damage.

## Data Availability

The original contributions presented in the study are included in the article/supplementary material, further inquiries can be directed to the corresponding author.
